# Delayed diagnosis of pheochromocytoma associated with chronic kidney disease

**DOI:** 10.4103/0971-4065.70843

**Published:** 2010-07

**Authors:** G. H. Fernandes, G. B. Silva Júnior, J. H. P. Garcia, C. R. M. Sobrinho, P. L. M. M. Albuquerque, A. B. Libório, E. F. Daher

**Affiliations:** Divisions of Nephrology and Cardiology, Hospital Universitário Walter Cantídio, School of Medicine, Universidade Federal do Ceará, Fortaleza, Ceará, Brazil

**Keywords:** Chronic kidney disease, hypertension, pheochromocytoma, surgery

## Abstract

Pheochromocytoma is a rare disease charactrized by excessive production of catecholamines, manifestating mainly with hypertension. We report the case of a 45-year-old woman with history of sudden onset dyspnea, headache, palpitations and sudoresis. An abdominal ultrasound was suggestive of chronic kidney disease (CKD). An abdominal computed tomography and magnetic resonance was performed and showed a mass in the topography of left adrenal. The patient underwent a surgery for the removal of the mass and became stable with normal blood pressure levels, but remained with CKD. The dalayed diagnosis of pheochromocytoma in the present case has contributed to the development of CKD.

## Introduction

Pheochromocytoma is a rare disease originating from medullary adrenal chromafin cells, characterized by excessive production of catecholamines.[1] The clinical manifestations range from unspecific symptoms to hypertensive emergencies.[2] The most common signs and symptoms are headache, palpitations, sudoresis, facial rash, tremors, syncope and weight loss. In 30% of cases, patients present with symptoms related to tumor compressive effect or the mass is incidentally found through an image exam.[3]

## Case Report

**Figure 1:**
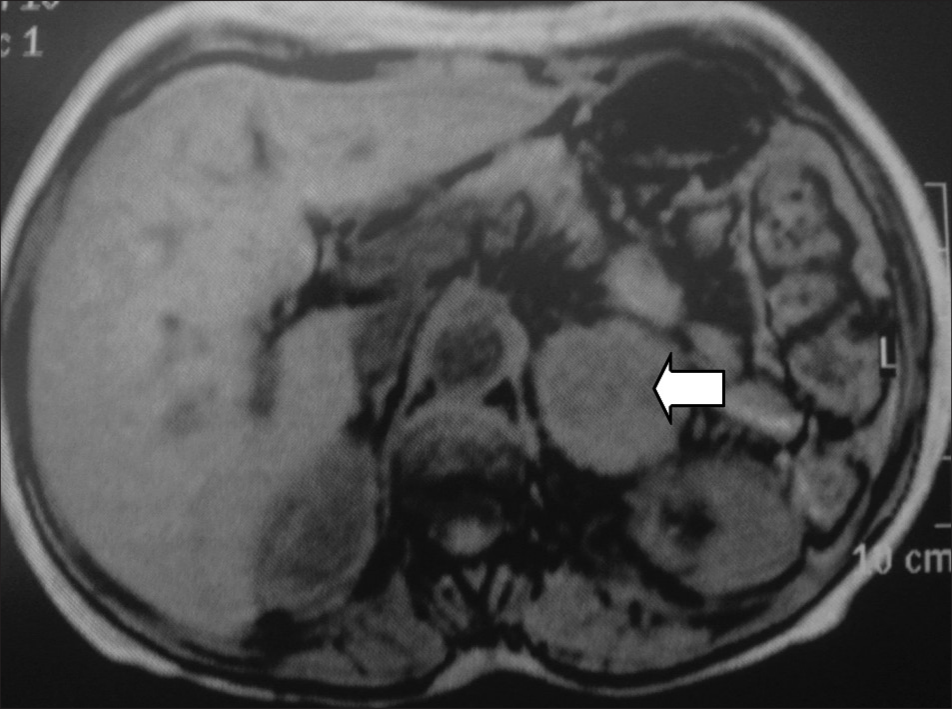
Nuclear magnetic resonance showing a mass in the left adrenal topography

**Figure 2:**
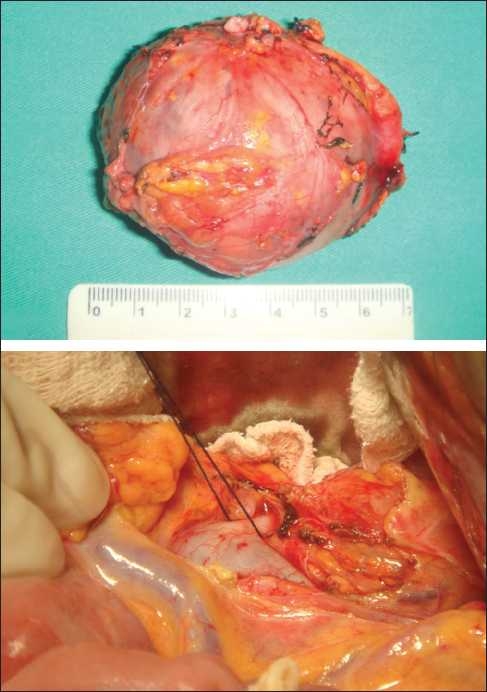
(a) Adrenal tumor and (b) adrenal vein

We report the case of a 45-year-old woman admitted to our hospital with a history of sudden onset dyspnea and hemoptysis. She also reported episodes of headache, palpitations and sudoresis, associated with weight loss. On physical examination, she had bilateral pulmonary crackles and blood pressure of 260/140 mmHg. Acute pulmonary edema was diagnosed and treatment with sodium nitroprusside and furosemide was suggested. Blood pressure was controlled and the patient became stable. Urinary metanephrines vanillyl mandelic acid was 25.7 mg/24 h (reference 2-14 mg/24 h), and metanephrines 519 µg/24 h (reference < 400 µg/24 h). Other laboratory test results are given in Table 1. An abdominal ultrasound was suggestive of chronic kidney disease (CKD) - right kidney measuring 9.0 × 4.0 × 5.4 cm, left kidney 9.0 × 4.2 × 4.0 cm, with no differentiation between cortex and medulla. Before hospital admission, the levels of creatinine were 1.6 and 1.8 mg/ dl. An abdominal computed tomography and magnetic resonance was performed and showed a mass shaped 4.6 × 5.0 cm in the topography of left adrenal [Figure 1]. The patient underwent surgery for the removal of the mass [Figure 2] and became stable with normal blood pressure levels, but remained with low eGFR. At hospital discharge, the eGFR was 37 ml/min. The patient is now in good clinical condition, with normal levels of blood pressure, without anti-hypertensive drugs, under conservative therapy for CKD.

**Table 1:** Laboratory test results during hospital stay

Day	Admission	1	2	6	9	13	16	17	18	20	22	32	33	35
Blood														
Hemoglobin (g/dl)	11.4	-	-	-	-	-	-	12.5	-	-	-	-	-	-
Hematocrit (%)	33.9	-	-	-	-	-	-	37.7	-	-	-	-	-	-
White blood cells (×10^3^/mm^3^)	12.1	-	-	-	-	-	-	6.59	-	-	-	-	-	-
Platelets (×10^3^/mm^3^)	4.47	-	-	-	-	-	-	2.53	-	-	-	-	-	-
Serum urea (mg/dl)	71	86	116	187	193	196	178	173	164	161	153	51	51	45
Serum creatinine (mg/dl)	2.0	2.6	3.1	3.1	3.2	3.2	3.2	3.3	2.5	2.6	2.6	1.6	2.0	1.4
eGFR* (mL/min)	26	20	17	17	16	16	16	16	21					37
Serum sodium (mEq/l)	143	139	138	135	135	135	140	138	139	138	137	138	137	141
Serum potassium (mEq/l)	4.0	3.7	3.8	4.4	4.1	4.0	4.5	4.5	3.7	4.3	4.5	3.6	4.6	5.7
Ionic calcium (mEq/l)	1.24	-	1.31	-	-	1.30	-	1.25	-	-	1.21	1.08	1.35	1.30
Total calcium (mg/dl)	-	-	-	-	-	-	-	-	7.8	-	10	-	-	9.6
Serum phosphorus (mg/dl)	-	-	-	-	-	-	7.1	-	6.0	3.9	4.0	-	-
Serum magnesium (mEq/l)	2.8	-	2,5	-	-	-	-	2.4	-	-	2.3	1.5	1.8	2.3
Urine														
Protein	-	-	-	-	+	-	-	-	-	-	-	-	-	-
Casts	-	-	-	-	-	-	-	-	-	-	-	-	-	-

* eGFR = Estimated glomerular filtration rate (In mL/min), according to Cockcroft-Gault formula

## Discussion

Pheochromocytoma is one of the most important endocrine causes of hypertension, followed by primary hyperaldosteronism and Cushing syndrome.[4] The annual incidence is around 2-8 cases per million people, with a world incidence of 0.05-0.12%.[5] The main complications of pheochromocytoma are myocarditis, arrhythmias, acute pulmonary edema, cardiogênic shock, stroke and even death. Chronic kidney disease (CKD) has not been described as a complication of pheochromocytoma. In the present case, the patient had a history of ten years with episodes of proxismal hypertension, which is suggestive of pheochromocytoma. In this patient, persistent hypertension could have contributed to the development of chronic renal insufficiency. Pheochromocytoma in a CKD patient has been described, but a cause-effect association has not been described.[4] The delayed diagnosis of pheochromocytoma in the present case may have contributed to the development of CKD.
